# Supplementary material for: Evaluation of a pharmacist-led actionable audit and feedback intervention for improving medication safety in UK primary care: An interrupted time series analysis
Source: PLoS Med. 2020 Oct 13;17(10):e1003286. doi: 10.1371/journal.pmed.1003286 (PMC7553336; doi:10.1371/journal.pmed.1003286)
Supplement: S1 Fig — Screenshots from the SMASH interactive dashboard (Figs A–D) and forest plots showing variation between practices in the reduction in potentially hazardous prescribing and inadequate monitoring (Figs E–J). (DOCX) [file pmed.1003286.s001.docx]

**Figure A.** Screenshot from the SMASH interactive dashboard showing the overview of indicators and practice-level summary statistics with benchmark information.

**
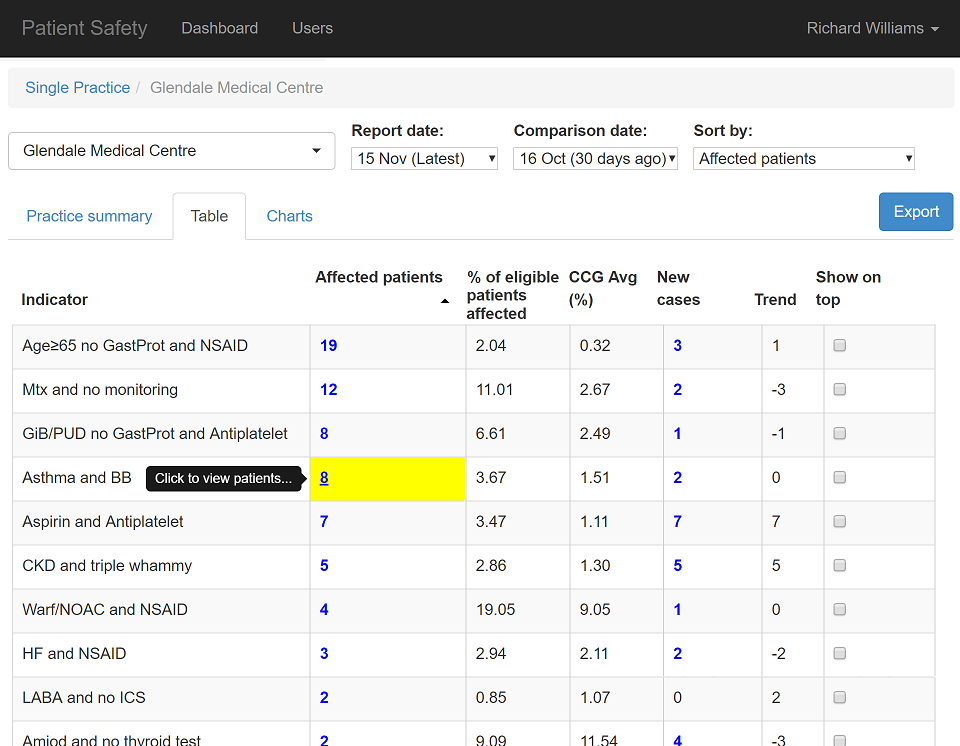
**

**Figure B.** Screenshot from the SMASH interactive dashboard showing a patient list for a specific indicator.

**
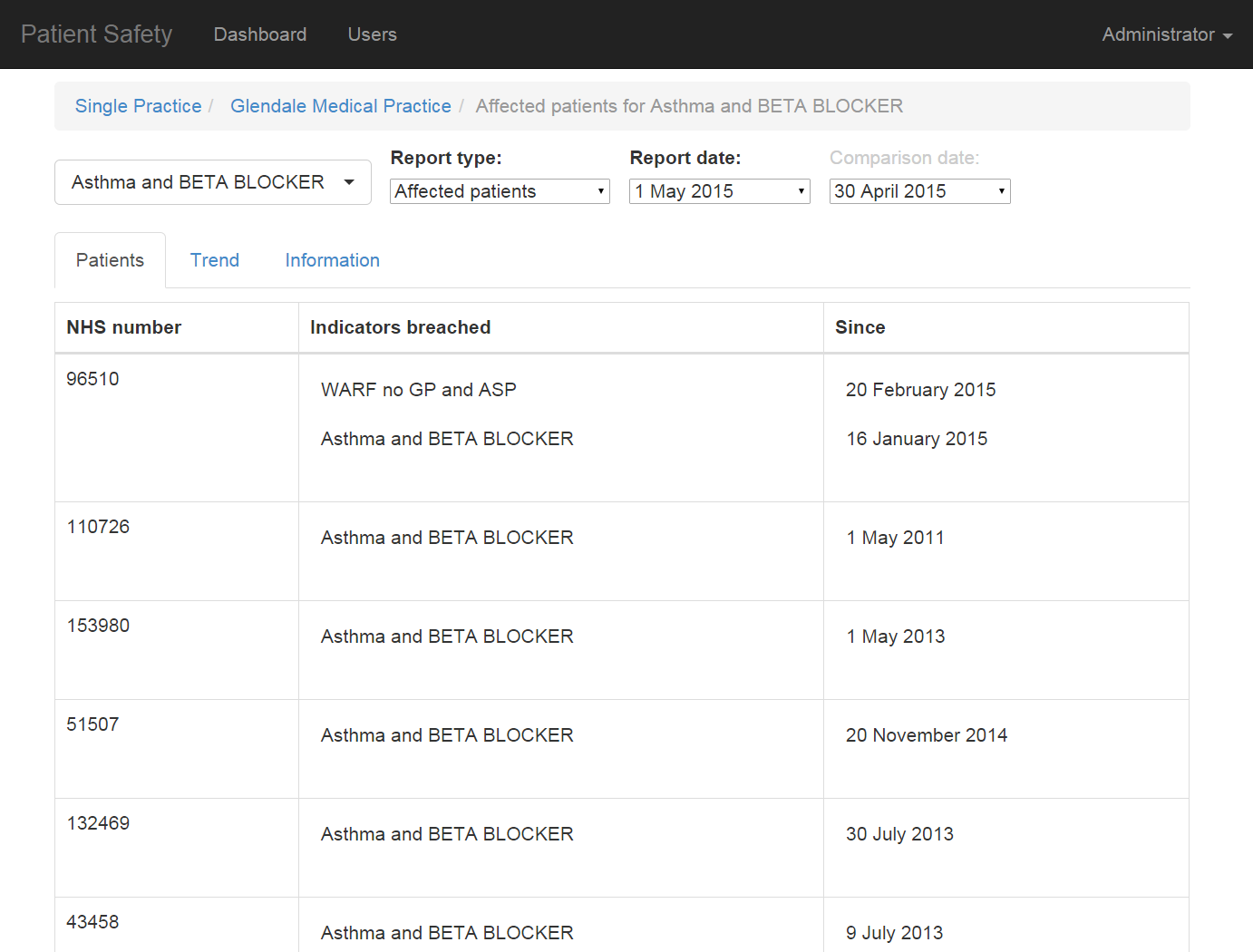
**

**Figure C.** Screenshot from the SMASH interactive dashboard showing educational material.

**
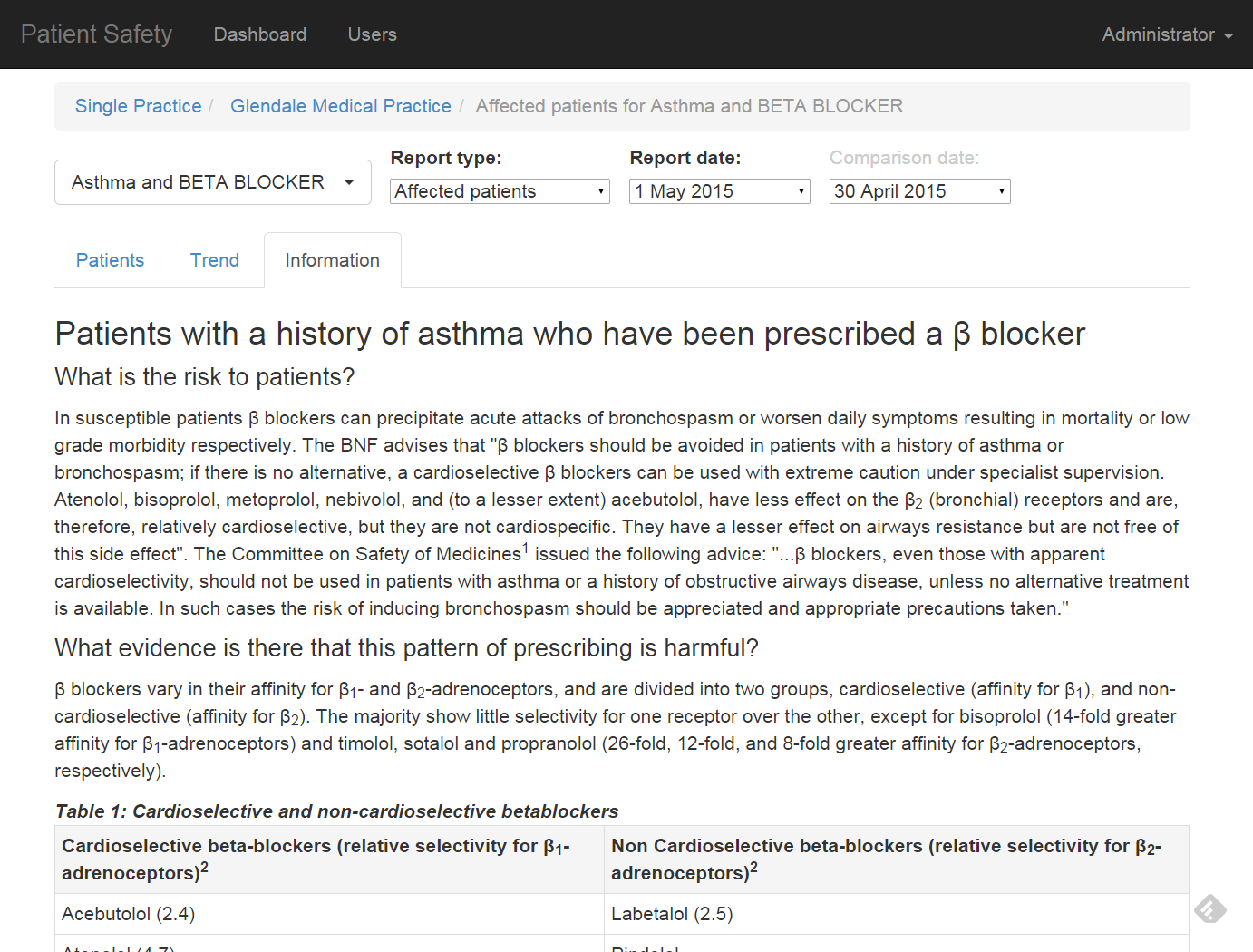
**

**Figure D.** Screenshot from the SMASH interactive dashboard showing a graph with practice-level trends over time.

**
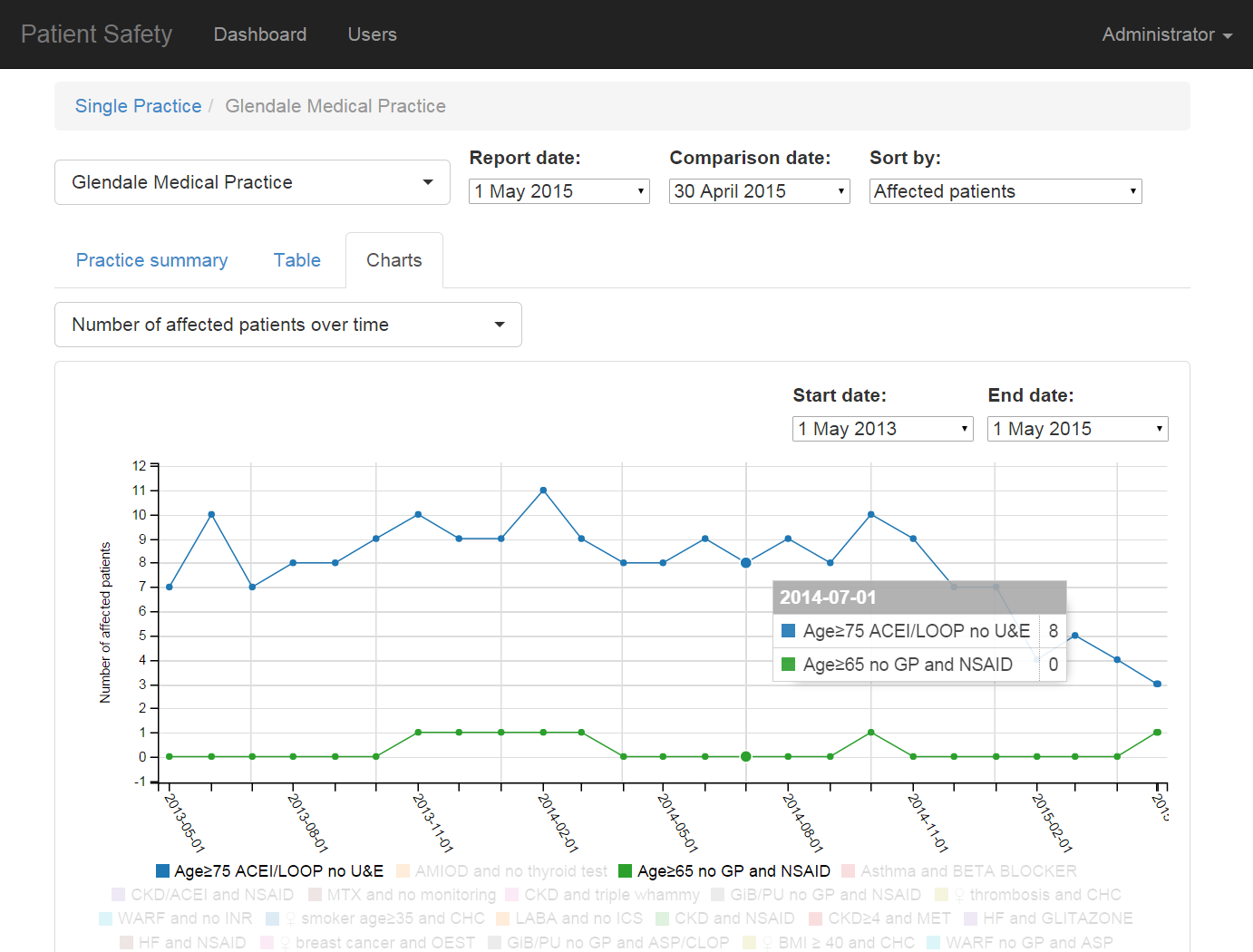
**

**Figure E.** Forest plot showing variation between practices in the reduction in potentially hazardous prescribing after 12 weeks.


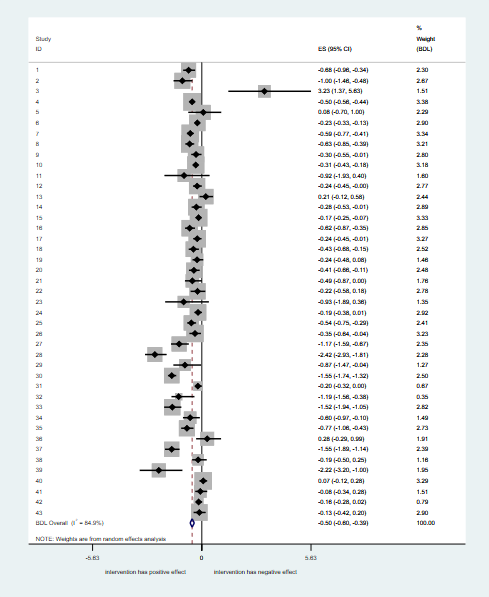


**Figure F.** Forest plot showing variation between practices in the reduction in potentially hazardous prescribing after 24 weeks.


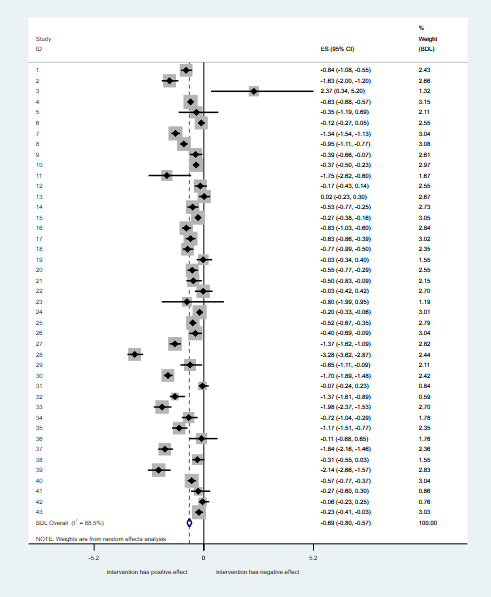


**Figure G.** Forest plot showing variation between practices in the reduction in potentially hazardous prescribing after 12 months.


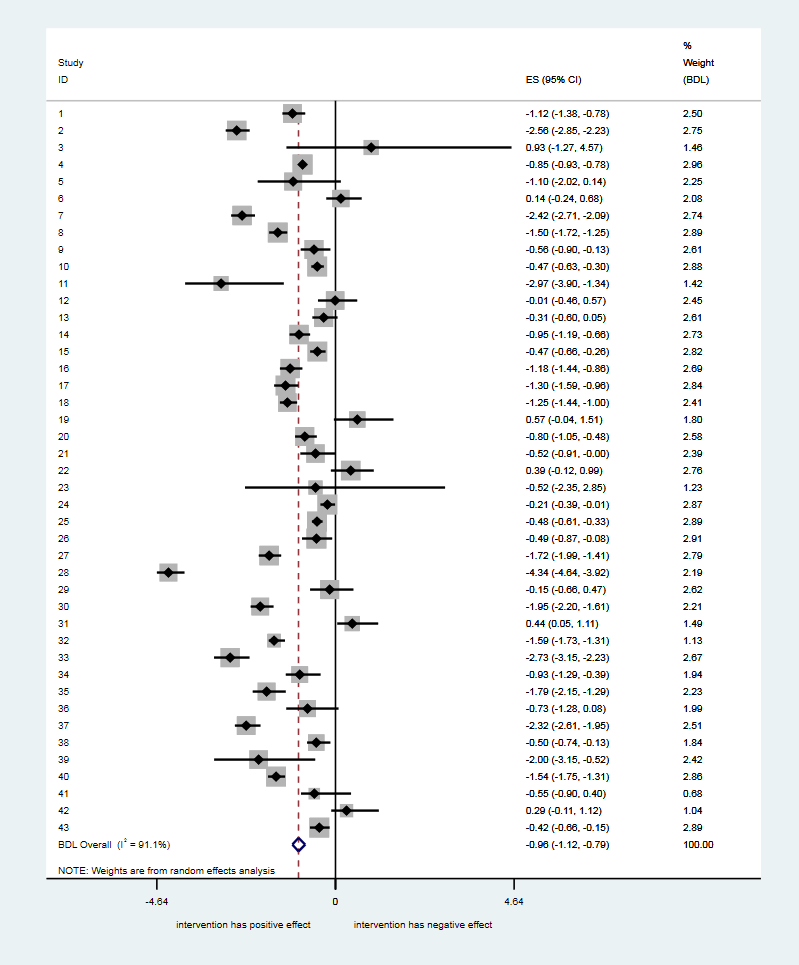


**Figure H.** Forest plot showing variation between practices in the reduction of inadequate blood-test monitoring after 12 week.

**
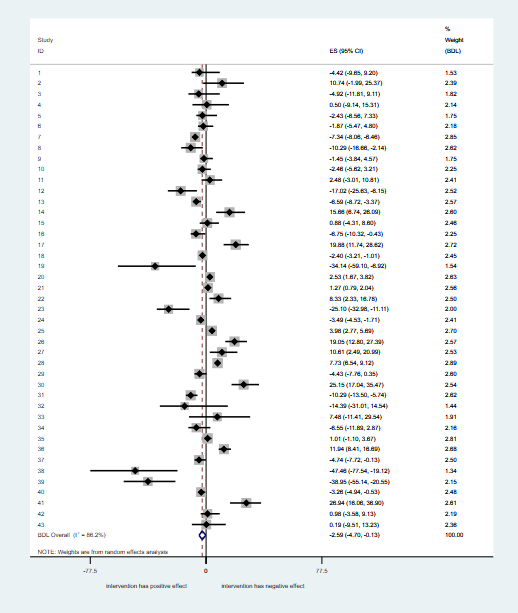
**

**Figure I.** Forest plot showing variation between practices in the reduction of inadequate blood-test monitoring after 24 weeks.

**
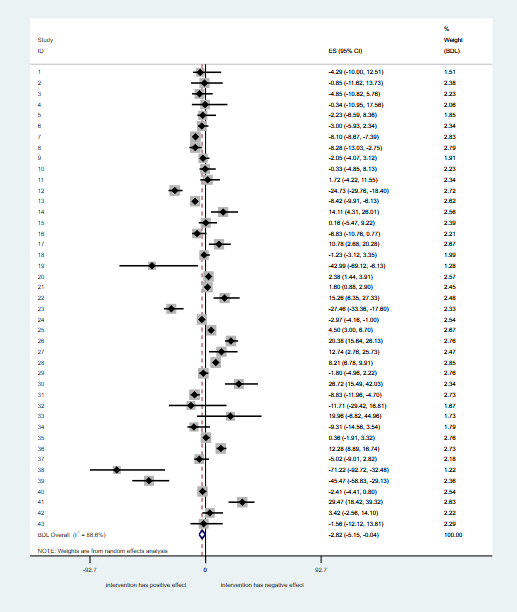
**

**Figure J.** Forest plot showing variation between practices in the reduction of inadequate blood-test monitoring after 12 months.

*
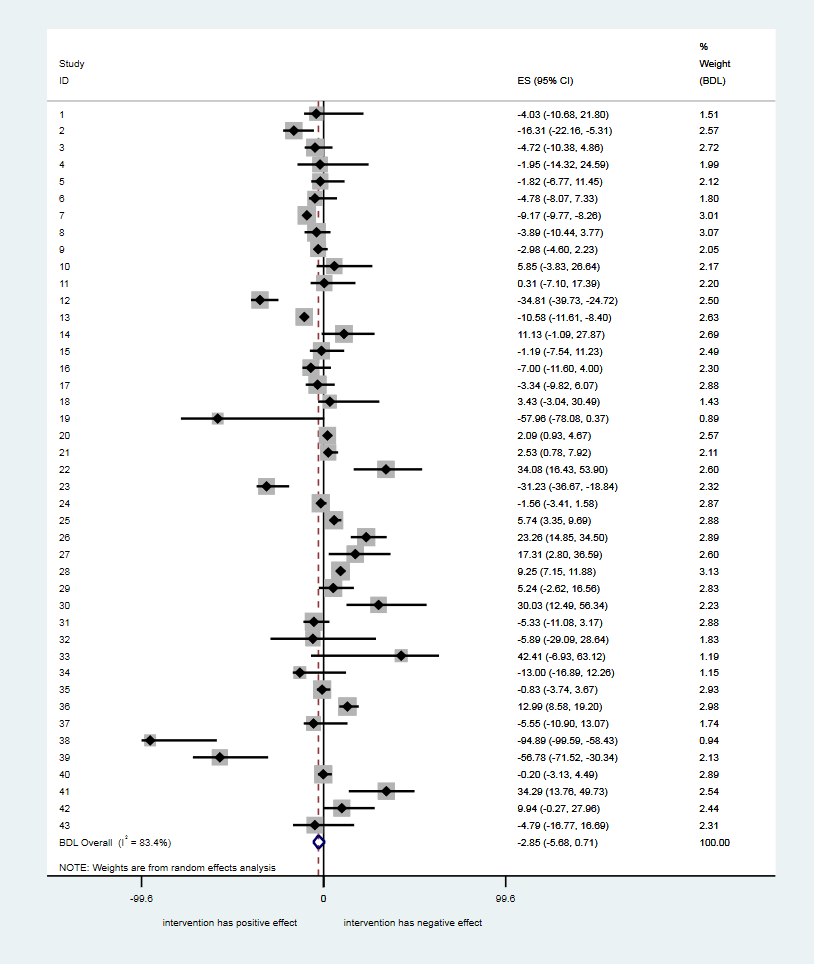
*
