# Supplementary material for: Evaluation of a pharmacist-led actionable audit and feedback intervention for improving medication safety in UK primary care: An interrupted time series analysis
Source: PLoS Med. 2020 Oct 13;17(10):e1003286. doi: 10.1371/journal.pmed.1003286 (PMC7553336; doi:10.1371/journal.pmed.1003286)
Supplement: S1 Study Protocol — (PDF) [file pmed.1003286.s002.pdf]

Research Protocol

The Salford MedicAtion Safety dasHboard (SMASH) Trial

Dr Richard N Keers, Mr Mark Jeffries, Dr Denham Phipps,
Dr Niels Peek, Dr Evangelos Kontopantelis, Mr Richard Williams,
Professor Darren M Ashcroft
(The University of Manchester)

Dr Sarah Rodgers, Professor Tony Avery
(The University of Nottingham)

Contents

1. BACKGROUND 3

2. THE 'SMASH' INTERVENTION 4

3. AIM AND OBJECTIVES........ 5

3.1 Principle study objectives........ 5

3.2 Secondary research objectives 5

4. FUNDING 5

5. METHODS 6

5.1 Quantitative Evaluation........ 6

5.2 Qualitative Process Evaluation 8

6. CONFIDENTIALITY 11

6.1 Quantitative Evaluation........ 11

6.2 Qualitative Process Evaluation 11

7. RISKS 12

7.1 Quantitative Evaluation........ 12

7.2 Qualitative Process Evaluation 12

8. BENEFITS 13

9. TIMESCALES........ 13

10. REFERENCES 14

APPENDICES

I. Cover Letter to General Practices

II. Participant Information Sheet for General Practices

III. Consent form for General Practices

IV. Interview schedule: Process Evaluation

V. Participant Information Sheets: Qualitative Process Evaluation

VI. Consent forms: Qualitative Process Evaluation

1. BACKGROUND

Medication management in primary care is becoming increasingly complex because more and more people use multiple types of medication, have several chronic diseases, or both. Considering the vast number of medication prescriptions issued in primary (more than 2.7 million in England each day),[1] it is perhaps not surprising that errors are being made during the prescribing and monitoring of medication by general practices.[2]

Increasingly, tools based on information technology are available to support general practitioners and other care professionals in their daily work. One of the tools that has recently emerged is the electronic dashboard. Dashboards provide feedback in the form of summary data on predefined metrics (indicators) and use data visualization techniques to help care professionals interpreting these summaries. One notable example is the recently launched NHS England Medicines Optimisation Dashboard (see http://www.england.nhs.uk/ourwork/pe/mo-dash/).

The increasing use of IT in healthcare has the potential to impact upon medication safety. IT interventions incorporating audit and feedback modalities may provide improved access to prescribing data and allow clinicians, and other health care professionals, to assess the quality and safety of prescribing for improvement purposes. Specifically, interrogation of Electronic Health Records (EHRs) using the audit and feedback approach may provide opportunities to identify and correct suboptimal performance and improve care,[3,4] which could include prescribing and monitoring errors, which may thus prevent harm to patients.

The landmark PINCER trial demonstrated how pharmacists working collaboratively with primary care physicians to act upon electronic medication error data presented in Quality Improvement (QI) dashboard software reduced the number of prescribing errors.[5] The PINCER pharmacists were trained in educational outreach and root cause analysis techniques so that they could identify, explore and resolve/prevent medication errors in partnership with practice staff.[6] Although the evaluation identified important factors which could underpin the successful implementation of PINCER in practice[7] exactly how the electronic feedback was accessed and used by stakeholders, and how they interacted with each other in this context was not thoroughly investigated. Whilst the PINCER trial emphasised the importance of regular face-to-face contact between stakeholders for improving medication safety, details as to the dynamics of such interactions and their connection to the QI software dashboard tool were not specified.

2. THE 'SMASH' INTERVENTION

Our SMASH intervention is based on the PINCER intervention that was approved by the Nottingham 2 research ethics committee in 2006, and was successfully evaluated in Nottinghamshire, Staffordshire, and central and eastern Cheshire.[5] Each general practice that provides consent for the study will receive this intervention, i.e. the electronic medication safety dashboard and visits by a clinical pharmacist. As with the PINCER study, the likely active component of this complex intervention is the pharmacist outreach visits; the dashboard primarily will support information delivery (like the control arm in PINCER). The main difference between our study and the PINCER trial is the use of an updated interactive electronic medication safety dashboard as the IT component of this intervention, containing a modified and updated set of prescribing and monitoring safety indicators.

Members of the project team have recently developed this updated interactive medication safety dashboard in collaboration with stakeholders (pharmacists, GPs and a patient).[8] The dashboard is designed to identify and feedback instances of potentially hazardous prescribing in a way which facilitates optimal use in primary care practice for improvement purposes.[8] The dashboard incorporates an updated and expanded list of prescribing safety indicators,[9] and interrogates patient care records to identify these medication safety problems within a practice, and helps to trace the patients involved. This dashboard used alongside clinical pharmacy support has the potential to fulfil a "safety net" function and reduce avoidable patient harm. In addition to the use of the updated electronic dashboard within SMASH, practices will be visited by a clinical pharmacist trained to deliver educational outreach similar to the PINCER trial intervention.[5,6]

3. AIM AND OBJECTIVES

We aim to evaluate the impact of a complex pharmacist-led information technology intervention in Salford (Greater Manchester) on the incidence of potentially hazardous prescribing and monitoring, as well as understanding how this intervention is implemented and used in general practice. The intervention combines access to an updated electronic medication safety dashboard containing modified prescribing safety indicators, along with outreach visits to general practices by trained clinical pharmacists to address safety issues identified by the dashboard.

3.1 Principle study objectives

a. To evaluate the effect of a complex pharmacist-led intervention with an updated electronic medication safety dashboard on medication safety in primary care.
b. To assess the relationship between usage patterns of an electronic dashboard and its effect on medication safety in primary care.
c. To understand how the pharmacist-led intervention is implemented and embedded into everyday practice, and how pharmacists and other stakeholders interact in different contexts to achieve outcomes using the electronic dashboard tool.

3.2 Secondary research objectives

a. What is the frequency with which the electronic dashboard is used, who are its primary users (pharmacists or practice staff), and how does this vary between practices and over time?
b. Which feedback modalities provided by the electronic dashboard (table, benchmark charts, trend charts, patient lists) are accessed by users, and under which circumstances?
c. On which areas of medication safety (i.e. which prescribing safety indicators) do users tend to focus when they access the electronic dashboard?
d. How does activity with the electronic dashboard relate to improvements in medication safety, as measured through the dashboard's indicators?
e. How is the intervention understood, implemented and used in everyday practice?
f. How do contextual factors such as work practices, socio-organisational structures and behaviours impact upon the way the intervention is achieved in everyday use?
g. How do different participants, including the pharmacist delivering the intervention and the GP staff, respond to and interact with the intervention?
h. How, if at all, is work practice adapted, changed and sustained in a process of normalizing the intervention into everyday practice?

4. FUNDING

The SMASH trial will be undertaken as part of ongoing research within the Greater Manchester Primary Care Patient Safety Translational Research Centre (GM PSTRC) to improve patient safety within primary care settings. The GM PSTRC is funded by the National Institute for Health Research (NIHR).

5. METHODS

5.1 Quantitative Evaluation

Setting

We will evaluate the SMASH intervention in Salford (Greater Manchester) Clinical Commissioning Group (CCG), which has a population of 250,000, using mixed methods. Each one of 47 individual general practices in Salford CCG will be eligible to participate in the study.

Sampling

Each one of 47 individual general practices in Salford CCG will be eligible to participate in the study. In those practices that participate, all GPs in the practice will get access to the electronic medication safety dashboard, and they will be visited by a clinical pharmacist who will assist in understanding and solving medication safety problems that have been identified with the dashboard.

For the quantitative evaluation, we assumed that there will be 20000 eligible patients (10000 before and 10000 after the intervention) in 47 Salford general practices. We assumed that 30 practices (64%) will participate in the study. The ipdpower command in Stata was used to estimate the power obtained, assuming a small level of heterogeneity for both the intervention and the intercept (the baseline level of medication errors across practices), which are realistic assumptions that greatly affect power calculations. The code is available from the authors. For the numbers quoted above power was estimated to be 88.3% with multi-lever regression modelling, to detect a drop in the probability of an adverse outcome from 0.060 (pre-intervention) to 0.045 (post-intervention in the 30 practices receiving the intervention).

General practices whose electronic health record system is not linked to the Salford Integrated Record database cannot participate in the study.

Participants

Our primary participants are GPs, other general practice staff such as nurses and managers, and pharmacists. Patients are indirect participants of the study. For the quantitative work will not recruit individual GPs but entire general practices because the intervention is applied at practice level. Any general practice in the city of Salford, Greater Manchester, is eligible for participation. When a general practice participates, this will imply that all their staff directly participate in the study (quantitative and qualitative observation elements) and all their patients indirectly participate in the study.

Study design

This study uses an experimental design, in which general practices receive the intervention at different points in time. We will evaluate the effect of the intervention quantitatively using an interrupted time series study design, by comparing the numbers of patients at risk of avoidable harm in each practice before and after the intervention through analysis of incident rates of prescribing and monitoring safety indicators identified by the medication safety dashboard. In addition, we will use log files of the electronic dashboard to investigate the frequency with which the electronic dashboard is used, who its primary users are (e.g. pharmacists or GPs), and how this varies between practices and over time. We will also analyse the log files to assess which feedback modalities provided by the electronic dashboard (table, benchmark charts, trend charts, patient lists) are typically accessed by users, and under which circumstances. Finally, we will analyses the log files to study which areas of medication safety (i.e. which indicators) users tend to focus when they access the electronic dashboard.

Time period

Upon entering the SMASH trial, practices will be given access to the dashboard and receive visits by the clinical pharmacists over 10 days during at least a 12 week intervention period. Following this period, the pharmacist will be removed but the dashboard will remain in place. It is anticipated that practices will enter the trial at

different time points in a naturalistic fashion. Following the 12 week intervention period, there will be a variable follow-up times after that, with a maximum of 15 months, during which time SMASH trial evaluation may continue.

Installation and access to the dashboard
A member of the research team (RW) will install the SMASH dashboard software on two servers at Salford Royal Foundation Trust (SRFT) data centre. This will be done remotely via a secure remote desktop connection to the servers. A feed of patient data from the Salford Integrated Record will be set up to allow the dashboard to be populated. No physical installation at practice level is necessary.

GPs and NHS employed clinical pharmacists will be able to access the SMASH medication safety dashboard through their web browser using a personal account (password protected) only on the Salford secure network. The dashboard is accessible 24/7 throughout the study and also can be accessed remotely using VPN connecting to the secure network.

Data collection method
We will evaluate the effect of the intervention quantitatively using an interrupted time series study design, by comparing the numbers of patients at risk of avoidable harm in each practice before and after the intervention through analysis of prevalence rates of prescribing and monitoring safety indicators identified by the medication safety dashboard. In addition, we will use log files of the electronic dashboard to investigate the frequency with which the electronic dashboard is used, who its primary users are (e.g. pharmacists or GPs), and how this varies between practices and over time. We will also analyse the log files to assess which feedback modalities provided by the electronic dashboard (table, benchmark charts, trend charts, patient lists) are typically accessed by users, and under which circumstances. Finally, we will analyse the log files to study which areas of medication safety (i.e. which indicators) users tend to focus when they access the electronic dashboard.

The primary outcome measures will be the number and proportion of patients in each general practice:
a) Exposed to at least one type of hazardous prescribing
b) Exposed to at least one prescription monitoring error

The secondary outcome measures will be:
c) The frequency of using the dashboard and length of time actively using the dashboard in each session, in each general practice
d) Patient factors and/or patient subgroups that are associated with the effectiveness of the intervention

Recruitment
General practices potentially suitable for recruitment will be identified through discussions between Salford CCG and local GP Quality Leads. The University of Manchester will receive a list with contact details of all general practices in Salford.

Practice managers/senior partners will then be directly approached by the CCG pharmacy teams (supported by the research team) and provided information (see Appendix I and II) about the study suitable for sharing amongst their colleagues, along the opportunity to take part. After providing this information, practices will be asked to return a tear away slip to The University of Manchester indicating their interest in taking part (see Cover Letter, Appendix I). Interested parties will then be contacted by the research team to obtain written consent (see Appendix III), with those practices not responding after 2-3 weeks being phoned by the research team to obtain levels of interest and consent. Practices who have questions about the study will be encouraged by these written communications and be the CCG pharmacy teams to contact the University researchers.

There will be no recruitment of patients, but GPs, pharmacists and general practice staff will have access to patient data through the Salford Integrated Record (SIR). SIR is a database that integrates data from all primary and secondary care providers in Salford. It does not include information on mental and sexual health. Researchers will have access to anonymised records from SIR for the quantitative evaluation.

We will obtain global written informed consent for all staff in each general practice from the practice manager/senior partner to undertake the quantitative and qualitative observation work (as well as general permission to approach practice staff for interview, but each of whom will be individually consented).

Patient and Public Involvement
Our study is carried out as part of the NIHR Greater Manchester Primary Care Patient Safety Translational Research Centre (NIHR GM PC PSTRC, 2013-2017). The Centre has a Research User Group (RUG) consisting of members of the public with an interest in patient safety in primary care. One of the Centre's RUG members, Ms. Faith Mann, will be actively involved in the study. She will be invited to meetings of the research team, attend several interviews with GPs and pharmacists, and participate in the qualitative analysis of interviews and observations to gather the patient and public perspective on these data.

Study Advisory Group
Ms. Mann (PPI representative) will also be part of the Study Advisory Group, which further consists of Dr. Sheila McCorkindale (Salford GP and local NIHR Clinical Research Network lead for Primary Care), Dr. Claire Vaughan (Head of Medicines Management, NHS Salford CCG), members of the research team and other IT and quality representatives (e.g. Salford GP quality leads (to be recruited)). The Study Advisory Group will support the implementation and optimization of the intervention in practice.

Data analysis
Quantitative data (extracts from the Salford Integrated Record and dashboard usage activity logs) will be analyzed at the safe data haven of the MRC Health e-Research Centre (HeRC) in Manchester.

Data will be archived in a durable form that is immune to subsequent tampering and falsification by the University of Manchester for at least 5 years before being destroyed. The data will be stored in a way that permits a complete retrospective audit if necessary. Apart from the Chief Investigator, only authorized personnel from the University of Manchester will have access to the data.

Multi-level logistic regression will be used to quantify the effect of the intervention at the patient level, within the interrupted time-series quasi-experimental design.[10] We will account for the nested structure of the data (patients within practices) and the fact that the intervention is applied at the practice level. Analyses will be controlled for relevant covariates (e.g. usage of the dashboard).

5.2 Qualitative Process Evaluation

Setting
The qualitative work will be undertaken in up to sixteen General Practices within Salford CCG, and will work in parallel to the implementation of the SMASH intervention and the quantitative analysis of the dashboard use.

Sampling
A range of General Practices across the CCG will be purposefully sampled to reflect the different contexts occurring from variations in practice size, mean patient age, social deprivation and use of patient record software (EMIS, VISION).

Participants
- Four pharmacists working for Salford CCG, who will carry out the SMASH intervention

- Up to thirty practice staff to include a full range of practice staff – GPs, nurses, and practice managers – at least one member of staff at each practice included in the observations

Time period
- The duration of the SMASH intervention period, plus approximately eight to twelve weeks

Theoretical framework
Interventions to improve health care are complex[11] because they involve a range of structures and a variety of stakeholders. Qualitative process evaluations can be of use in unpicking this complexity.[11] In this study it is assumed that the intervention will be dependent on a range of social factors including institutional work practices, hierarchies and divisions of labour, structure and agency.[12]

We will seek to explore the complex social interactions within practices where the intervention is implemented and the dynamics between a range of stakeholders and the electronic dashboard tool and unpick the contextual factors that might impact upon the intervention.[11,13] It will be of value to see how the intervention works within the pre-existing workflow of the practices and the clinicians involved.[14] We will also look to explore the changes in work practices within general practices once the PINCER pharmacist intervention period has passed in order to better understand how to maximise sustainability of this intervention. To achieve this, the qualitative evaluation draws upon two complementing theoretical frameworks previously utilised in understanding and evaluating interventions:

Normalization process theory (NPT) can highlight the ways in which an intervention is integrated and adopted into everyday practice. This has particular utility in examining how individuals and groups understand the intervention through processes of sense-making and how the work that enables the intervention to happen.[15,16] A Realist Evaluation does not attempt to apply a simple cause and effect interpretation of an intervention but seeks to explain the ways the intervention might work, for whom and in what circumstances.[17,18] The outcomes from an intervention are seen to be dependent on particular responses and actions from individuals and groups (mechanisms) activated in particular existing social and cultural circumstances (contexts).[17,19] It is has been argued that process evaluations need to consider the fidelity and quality of implementation, understand the mechanisms associated with how and why the intervention might lead to outcomes and to identify contextual factors.[20] It is thought that NPT would be an ideal framework for understanding what is delivered, how implementation is achieved within different contexts and the ways in which the intervention might be sustained in everyday practice. Realistic evaluation would be ideal for examining mechanisms of impact within specific contexts that would explain how the intervention worked. This might be of particular value considering the range of practices and that different pharmacists will be involved.

Data collection method
We expect to carry out up to sixteen non-participant direct observations with the four pharmacists implementing the SMASH intervention at up to four practices each. Each observation period is expected to last between four to eight hours. Observations will be recorded using contemporaneous researcher field notes. Following the observation periods we will conduct face-to-face semi-structured interviews with the pharmacists resulting in up to sixteen interviews in total. Up to thirty further interviews (depending on the number of practices recruited) will be conducted with practice staff who have received the intervention, either face-to-face or by telephone (GPs, practice managers and practice nurses) including at least one member of staff at each practice included in the observations. Each interview will be approximately forty minutes in duration and participants will be asked for the interviews to be digitally audio-recorded for transcription. The interviews will take place in private at the participant's usual place of work or other convenient venue of their choosing. The interviews will illicit views about (see Interview schedule, Appendix IV):

-how the intervention is conceptualized and understood by participants

-the expectations and experiences of the intervention
-the ways in which the dashboard has been used in the practice
-the benefits and drawbacks of the intervention
-the circumstances under which the intervention has proved most effective

Approximately eight to twelve weeks following the completion of the SMASH intervention period, further interviews will take place with up to thirty staff (nurses, GPs, managers) from practices participating in the observations – with at least one member of staff per practice as above. These will be conducted by telephone, will last approximately twenty minutes and be digitally audio-recorded for transcription. These interviews will explore any changes in work practices within general practices once the pharmacist intervention period has passed. The observations and interviews will be conducted by a researcher trained in qualitative research (MJ). Interviews will continue until the maximum number reached or saturation of themes is observed.

Recruitment
Recruitment will combine with the roll-out of the SMASH intervention with approximately a third of the practices involved in the intervention sampled for the qualitative evaluation based on the criteria above to achieve a diverse range of practices. The research team (MJ, RNK, NP, DP, DMA) will meet with the CCG pharmacists and GP quality leads to provide information about the study and explore conduits for recruitment. It is hoped to utilise the quality leads and pharmacists to help identify practices interested in taking part. Once participating surgeries have been identified MJ will contact practice managers at participating surgeries to identify surgery staff interested in taking part in interviews. Staff may also be approached for interview during the pharmacist observations. These staff will then be given information about the study (see Appendix V) and invited to participate before being contacted to arrange interviews. The CCG pharmacists will be verbally consented at an early stage and then will give written informed consent before each observation and interview (see Appendix VI). Practice staff will give informed consent at the commencement of the interviews (see Appendix VI). Observations of practice staff will be made as part of the pharmacist observations, and consent will be provided at practice level through managers/senior partners (See Appendix III).

Participants will be refunded for the loss of their time as a result of taking part in interviews. In line with advice from NIHR North West Primary Care Research Network, GPs, Pharmacists and general practice staff will be compensated as follows: GPs £40 per hour; general practice staff and pharmacists £20 per hour.

Data management
Study observation data will be transcribed into electronic form and then kept on a secure server at the University of Manchester. Interview audio files will be transcribed using an approved university transcribing service (files transferred using secure link) and stored on a secure password protected electronic server. This server is accessible only to those with login credentials. Consent forms will be kept in hard copy at a secure location away from the study site. Data will be stored separately from the documentation matching participant numbers with personal details.

Data analysis
Analysis will be iterative and concurrent with data collection. Initial exploration of the data will allow for emerging themes to be explored in subsequent interviews and observations. This will allow for data collection to end once saturation of themes has been reached. Analysis will be led by MJ. Emerging themes will be developed into coding frameworks and discussed across the research team. MJ will initially code all transcripts. RNK and DP will each then independently code a proportion of those transcripts. Emerging themes and codes will then be discussed by MJ, DP and RNK with DMA resolving differences.

6. CONFIDENTIALITY

6.1 Quantitative Evaluation

Our intervention discloses patient identities to practice staff (e.g. GPs) and pharmacists who treat them, which is not an issue as they would have access to this information as per their professional role. All data for the quantitative analysis will be pseudo-anonymised prior to the analysis at patient-level, GP-level, pharmacist-level and general practice-level.

The SMASH electronic medication safety dashboard will query the SIR database which stores NHS numbers of patients registered in general practices in Salford. Therefore the system will be hosted on secure servers from Salford Royal NHS Foundation Trust. GPs will be able to see the NHS numbers of patients registered in their practice for whom a safety hazard has been identified. This information will be transferred to their web browser through the secure N3 network. Our software engineers potentially have access to NHS numbers when they service the electronic dashboard system. However the NHS numbers are encrypted and held in a separate database to the anonymised identifiers and the rest of the data, to eliminate the possibility of accidentally accessing these NHS numbers. To prevent engineers maliciously accessing the NHS numbers they will sign an information confidentiality agreement as part of their honorary contract at Salford Royal NHS Trust, which will host the system, and always enter the server room in pairs.

Quantitative dashboard usage data for the study will be obtained by querying electronic activity logs of GP and pharmacist activity with the database. These logs will not contain patient data. Practice staff (e.g. GPs) and pharmacists will be identifiable in the logs.

The dashboard will be hosted on secure servers at the IM&T Datacentre from Salford Royal NHS Foundation Trust. The following physical security arrangement are in place to restrict access to the datacentre:
· Only approved IM&T Technical staff have access to the datacentre. ID Badge requirement with formal provisioning and approval process.
· 24x7 security guard and visitor identity verification with in-facility escort
· Electronic access control using badge/access card.
· Leaver personnel removed from facility access within 24 hours of notification, or sooner.
· Camera surveillance (CCTV) for critical areas and perimeter intruder alarm system
· No windows or other perimeter access points in sensitive areas (e.g. computer room).
· Security systems supported with uninterruptible power supply (UPS) for continuous operation.
· Receiving/delivery of hardware and other equipment verified by personnel.

6.2 Qualitative Process Evaluation

All staff will participate anonymously. All NHS professionals involved in interviews and observations will be specifically requested not to disclose any patient identifiable information during the interviews (e.g. by referring to patients' names). The observations will be exclusively of interactions between staff and the pharmacist. No observations will take place with the pharmacist or other staff where NHS numbers or other patient identifiers are visible on the computer screen. In such circumstances where this is likely the pharmacist will be requested to turn off the screen prior to the observation. Each participant in the qualitative study will be assigned an identification number. This number, rather than the participant's personal details, will be used to identify any data associated with the participant. Anonymous quotations from participant's interviews or verbatim quotes from the field notes may be used in any reports, conference proceedings or publications.

Interview audio files, transcripts and any contact details of participants in the qualitative process evaluation will be kept on a password protected file on a University computer only accessible by the researcher. Consent forms will be kept in secure filing cabinets within the University and will be accessible only by the researchers.

7. RISKS

7.1 Quantitative Evaluation

Our intervention does not expose patients to any medical risks that would not exist otherwise. The intervention targets prescribing and monitoring behaviour of GPs; there is no intervention at patient level. Participating GPs are encouraged to adhere to an expanded list of medication safety indicators, which are based on thorough analysis of the latest literature by a panel of academic pharmacologists, GPs, and epidemiologists.[5] None of these indicators depart from recommendations in prevailing clinical practice guidelines. In addition, it will be emphasized to participating GPs that adherence to the indicators should not replace clinical judgement.

Any decision regarding the initiation, continuation or discontinuation of medication, as well as any other clinical decision, will only be made by the GPs or other appropriate practice staff (i.e. not the clinical pharmacists).

The electronic dashboard is accessed through a standard Web browser using a secure personal login with password. There is no need to install software on general practice computers, and therefore there are no IT security risks associated with using the dashboard.

7.2 Qualitative Process Evaluation

Medication errors are a potentially emotive topic we will therefore have contact details for local occupational health / other counselling support services to hand during interviews and observations. Participants will have the right to withdraw from interviews at any time. The researcher will be prepared to turn off the digital recorder and discontinue interview/observation if required.

There is a possibility that participants may divulge or the observer may see practices that may place patients/staff/public at risk of harm or cause harm. In these cases, incidents must be reported using local incident reporting systems and/or to line management as appropriate. If the participant refuses to self-report then the researcher will report on their behalf. All acts of purposeful negligence / criminal activity will be immediately reported by the researcher to local management. The researcher (MJ) is aware of the university policy on lone workers and will adhere to those guidelines.

During interviews, participants may be asked to discuss their own prescribing errors and interactions with other members of staff which may involve altercations or disagreements; these may be potentially sensitive depending on their nature. Participants will be reminded that all interviews are confidential and anonymous, and if they happen to mention people or places by name that these will subsequently be removed from the interview transcript. If required, the interviewer will turn off the audio-recorder and provide details of relevant professional support organizations if required (e.g. local counselling services).

Participation in qualitative interviews may create an additional time burden for participants. In order to prevent any undue burden we will emphasize to participants that their participation is voluntary and that they can withdraw from the study at any time with no penalty. Total participation time in up to 2 interviews has

also been limited to around 1 hour. Qualitative observation will take place whilst staff carry out their usual duties and is not expected to impinge on their time.

The researcher undertaking observations and interviews for the qualitative process evaluation will be working alone. This risk has been assessed by using a lone worker checklist and agreed to be minimal. The researcher will be undertaking lone worker training and operate in accordance with The University of Manchester lone working policy.

8. BENEFITS

For GPs, participation in the study will allow quick and easy identification of patients to whom potentially hazardous medication has been prescribed, or whose medication may not have been properly monitored. In addition, they will receive support (including visits) from a pharmacist in understanding and solving these issues.

For pharmacists, participation in the study will help to reduce the number of potentially hazardous medication prescriptions and improve medication monitoring. The study will also facilitate better collaboration with GPs in improving patient care.

The pharmacists, GPs and GP staff involved in the qualitative work will benefit from learning and understanding why new ways of working might succeed or fail and facilitating better health care team integration.

For patients, if their general practice participates in the study this will likely reduce the risk of adverse, medication-related events such as acute kidney injury and gastro-intestinal bleeds.

9. TIMESCALES

Start date: 1st October 2015

End date: 31st March 2017

All timescales below are approximate

Recruitment of pharmacists: October – November 2015

Recruitment of general practices: October 2015 – September 2016

Implementation of SMASH in practices: December 2015 – November 2016

Qualitative Process Evaluation: December 2015 – December 2016

Gathering/analysis of quantitative data: October 2015 – March 2017

Writing up/dissemination of results: September 2016 – end of 2017

10. REFERENCES

1. Prescribing and Primary Care Services. Prescriptions Dispensed in the Community, Statistics for England – 2002-2012. *Health and Social Care Information Centre*; 2013. Available from URL: http://www.hscic.gov.uk/catalogue/PUB11291/pres-disp-com-eng-2002-12-rep.pdf [Last accessed: 30/07/2015].
2. Avery AJ, Ghaleb M, Barber N, *et al.* The prevalence and nature of prescribing and monitoring errors in English general practice: a retrospective case note review. *Br J Gen Pract* 2013; DOI: 10.3399/bjgp13X670679.
3. Ivers N, Jamtvedt G, Flottorp S, *et al*. Audit and feedback: effects on professional practice and healthcare outcomes. *Cochrane Database Syst Rev* 2012; 6: CD000259.
4. Grimshaw JM, Thomas RE, MacLennan G, *et al*. Effectiveness and efficiency of guideline dissemination and implementation strategies. *Health Technol Assess Winch Engl* 2004; 8: iii–iv, 1–72.
5. Avery AJ, Rodgers S, Cantrill JA, *et al.* A pharmacist-led information technology intervention for medication errors (PINCER): a multicentre, cluster randomised, controlled trial and cost-effectiveness analysis. *The Lancet* 2012; 379: 1310-19.
6. Sadler S, Rodgers S, Howard R, *et al*. Training pharmacists to deliver a complex information technology intervention (PINCER) using the principles of educational outreach and root cause analysis. *International Journal of Pharmacy Practice* 2014; 22: 47-58.
7. Cresswell KM, Sadler S, Rodgers S, *et al.* An embedded longitudinal multi-faceted qualitative evaluation of a complex cluster randomized controlled trial aiming to reduce clinically important errors in medicines management in general practice. *Trials* 2012; 13: 78 doi: 10.1186/1745-6215-13-78
8. Keers RN, Williams R, Davies C, *et al*. Improving medication safety in primary care: developing a stakeholder-centred electronic prescribing safety indicator dashboard. *Pharmacoepidemiology and Drug Safety* 2015; Online Supp. DOI: 10.1002/pds.3812.
9. Spencer R, Bell B, Avery AJ, *et al*. Identification of an updated set of prescribing-safety indicators for GPs. *Br J Gen Pract* 2014; DOI:10.3399/bjgp14X677806.
10. Kontopantelis E, Doran T, Springate DA, *et al.* Regression based quasi-experimental approach when randomisation is not an option: interrupted time series analysis. *BMJ* 2015; 350: h2750.
11. Grant A, Treweek S, Dreischulte T, *et al.* Process evaluations for cluster-randomised trials of complex interventions: a proposed framework for design and reporting. *Trials* 2013; 14:15 doi.org/10.1186/1745-6215-14-15.
12. Wand T, White K, Patching J. Applying a realist(ic) framework to the evaluation of a new model of emergency department based mental health nursing practice. *Nurs Inq* 2010; 17: 231-239.
13. Oakley A, Strange V, Bonell C, *et al*. Process evaluation in randomised controlled trials of complex interventions. *BMJ* 2006; 332: 413-416.

14. Ranji SR, Rennke S, Wachter RM. Computerised provider order entry combined with clinical decision support systems to improve medication safety: a narrative review. *BMJ Qual Saf* 2014; 0: 1–8.
15. Murray E, Treweek, S, Pope C, *et al.* Normalisation process theory: a framework for developing, evaluating and implementing complex interventions. *BMC Medicine* 2010; 8: 63.
16. May C, Finch T. Implementing, Embedding and Integrating Practices: An Outline of Normalization Process Theory. *Sociology* 2009; 43(3): 535-554.
17. Wand T., White K., Patching J. Realistic evaluation of an emergency department-based mental health nurse practitioner outpatient service in Australia. *Nursing and Health Sciences* 2011; 13: 199-206;
18. Pawson R, Tilley N. *Realistic Evaluation.* London: SAGE, 1997.
19. Dalkin SM, Jones D, Lhussier M *et al.* Understanding integrated care pathways in palliative care using realist evaluation: a mixed methods study protocol. *BMJ Open* 2012; 2: e001533 doi:1136/bmjopen-2012-001533.
20. Moore G, Audrey S, Barker M *et al Process evaluations of complex interventions.* UK Medical Research Council (MRC), 2014.
