# Supplementary material for: Evaluation of a pharmacist-led actionable audit and feedback intervention for improving medication safety in UK primary care: An interrupted time series analysis
Source: PLoS Med. 2020 Oct 13;17(10):e1003286. doi: 10.1371/journal.pmed.1003286 (PMC7553336; doi:10.1371/journal.pmed.1003286)
Supplement: S1 Tables — Table A. Definitions of the medication safety indicators targeted by the SMASH intervention. Table B. Rates of hazardous prescribing and inadequate medication monitoring for 43 general practices in Salford. (ZIP) [file pmed.1003286.s003.zip › S1_Tables revised/S1 Table B.docx]

**S1 Table B.** Rates of hazardous prescribing and inadequate medication monitoring for 43 general practices in Salford.

| Outcome measure | Medication prescribing and monitoring hazard rates at 12 months before intervention start, baseline, and 12 weeks, 24 weeks, and 12 months after intervention start* | | | | |
| --- | --- | --- | --- | --- | --- |
|  | 12 months before start | Baseline | 12 weeks after start | 24 weeks after start | 12 months after start |
| Any prescribing hazard composite (1-11) | 1392/46374 (3.00) | 1230/47183 (2.61) | 1014/47364 (2.14) | 864/47289 (1.83) | 756/47163 (1.60) |
| Ongoing prescribing hazards** | 1134/46374 (2.44) | 1032/47183 (2.19) | 834/47364 (1.76) | 680/47289 (1.44) | 587/47163 (1.24) |
| New prescribing hazards | 258/46374 (0.56) | 198/47183 (0.42) | 180/47364 (0.38) | 184/47289 (0.39) | 169/47163 (0.36) |
| 1. Age 65+ no GastProt and NSAID | 594/20701 (2.87) | 472/20746 (2.28) | 358/20782 (1.72) | 280/20621 (1.36) | 219/20424 (1.07) |
| 2. GiB/PUD no GastProt and NSAID | 29/1405 (2.06) | 18/1407 (1.28) | 24/1370 (1.75) | 19/1350 (1.41) | 17/1303 (1.30) |
| 3. GiB/PUD no GastProt and Antiplatelet | 126/1405 (8.97) | 111/1407 (7.89) | 85/1370 (6.20) | 68/1350 (5.04) | 52/1303 (3.99) |
| 4. Warf/NOAC and NSAID | 58/3130 (1.85) | 44/3545 (1.24) | 44/3655 (1.20) | 41/3685 (1.11) | 44/3920 (1.12) |
| 5. Warf/NOAC no GastProt and Antiplatelet | 75/1837 (4.08) | 72/2096 (3.44) | 67/2150 (3.12) | 41/2125 (1.93) | 42/2214 (1.90) |
| 6. Aspirin and Antiplatelet | 162/4742 (3.42) | 144/4319 (3.33) | 107/4168 (2.57) | 85/4019 (2.11) | 74/3734 (1.98) |
| 7. Asthma and BB | 275/22745 (1.21) | 314/23276 (1.35) | 272/23361 (1.16) | 272/23383 (1.17) | 242/23367 (1.04) |
| 8. LABA and no ICS | 43/314 (13.69) | 38/277 (13.72) | 35/267 (13.11) | 32/255 (12.55) | 35/233 (15.02) |
| 9. HF and NSAID | 60/2394 (2.51) | 37/2523 (1.47) | 39/2558 (1.52) | 32/2551 (1.25) | 41/2586 (1.59) |
| 10. CKD and NSAID | 46/1906 (2.41) | 33/2107 (1.57) | 34/2176 (1.56) | 21/2164 (0.97) | 28/2182 (1.28) |
| Any monitoring hazard composite (12-13) | 98/653 (15.01) | 89/668 (13.32) | 55/658 (8.36) | 45/668 (6.74) | 61/652 (9.36) |
| Ongoing monitoring hazards** | 72/653 (11.03) | 71/668 (10.63) | 33/658 (5.02) | 27/668 (4.04) | 38/652 (5.83) |
| New monitoring hazards | 26/653 (3.98) | 18/668 (2.69) | 22/658 (3.34) | 18/668 (2.69) | 23/652 (3.53) |
| 12. Mtx and no monitoring | 37/456 (8.11) | 37/466 (7.94) | 29/468 (6.20) | 24/473 (5.07) | 29/471 (6.16) |
| 13. Amiod and no thyroid test | 61/198 (30.81) | 52/203 (25.62) | 27/192 (14.06) | 22/197 (11.17) | 32/183 (17.49) |

* Data are the total number of patients across practices, at each of the presented time points relative to the start of the intervention, who had risk factors (denominator) or were exposed (numerator) to potentially hazardous prescribing or inadequate blood-test monitoring.

** Ongoing high-risk prescribing was defined as the patient being exposed during previous and current measurement period to potentially hazardous prescribing or inadequate blood-test monitoring.
