# Supplementary material for: Evaluation of a pharmacist-led actionable audit and feedback intervention for improving medication safety in UK primary care: An interrupted time series analysis
Source: PLoS Med. 2020 Oct 13;17(10):e1003286. doi: 10.1371/journal.pmed.1003286 (PMC7553336; doi:10.1371/journal.pmed.1003286)
Supplement: S1 Tables — Table A. Definitions of the medication safety indicators targeted by the SMASH intervention. Table B. Rates of hazardous prescribing and inadequate medication monitoring for 43 general practices in Salford. (ZIP) [file pmed.1003286.s003.zip › S1_Tables revised/S1 Table A.docx]

**S1 Table A.** Definitions of the medication safety indicators targeted by the SMASH intervention.

| # | Description | Group exposed (numerator) | Group having risk factors (denominator) |
| --- | --- | --- | --- |
| Prescribing safety indicators | | | |
| Preventing gastrointestinal bleeding | | | |
| P1 | Prescription of an oral NSAID without co-prescription of an ulcer-healing drug^5^ in a patient aged ≥65 years. | Patients prescribed at least one oral NSAID within the 3 months leading up to the audit date. | Patients aged ≥ 65 years on the audit date without prescription of an ulcer-healing drug^5^ within the 3 months leading up to the audit date. |
| P2 | Prescription of an oral NSAID without co-prescription of an ulcer-healing drug^5^ to a patient with a history of peptic ulceration^4^. | Patients prescribed an oral NSAID within the 3 months leading up to the audit date. | Patients aged ≥ 18 years on the audit date with a history of peptic ulceration^4^ at least 3 months before the audit date without co-prescription of an ulcer-healing drug^5^ within the 3 months leading up to the audit date. |
| P3 | Prescription of an antiplatelet drug^2^ without co-prescription of an ulcer-healing drug^5^ to a patient with a history of peptic ulceration^4^. | Patients prescribed an antiplatelet drug^2^ within the 3 months leading up to the audit date. | Patients aged ≥ 18 years on the audit date with a history of peptic ulceration^4^ at least 3 months before the audit without co-prescription of an ulcer-healing drug^5^ within the 3 months leading up to the audit date. |
| P4 | Prescription of warfarin or NOAC^1^ in combination with an oral NSAID. | Patients prescribed an oral NSAID within the 3 months leading up to the audit date. | Patients aged ≥ 18 years on the audit date prescribed warfarin or NOAC^1^ within the 3 months leading up to the audit date. |
| P5 | Prescription of warfarin or NOAC^1^ in combination with and an antiplatelet drug^2^ without co-prescription of an ulcer-healing drug^5^. | Patients prescribed an antiplatelet drug^2^ within the 3 months leading up to the audit date and within 28 days of the prescription for Warfarin or NOAC^1^. | Patients aged ≥ 18 years on the audit date prescribed warfarin or NOAC^1^ within the 3 months leading up to the audit date without co-prescription of an ulcer-healing drug^5^ within the 3 months leading up to the audit date. |
| P6 | Prescription of aspirin in combination with another antiplatelet drug^3^ without co-prescription of an ulcer-healing drug^5^. | Patients prescribed another antiplatelet drug^3^ within the 3 months leading up to the audit date and with 28 days of the prescription for aspirin. | Patients aged ≥ 18 years on the audit date prescribed aspirin within the 3 months leading up to the audit date without co-prescription of an ulcer-healing drug^5^ within the 3 months leading up to the audit date. |
| Preventing exacerbation of asthma | | | |
| P7 | Prescription of a non-selective beta-blocker to a patient with asthma. | Patients prescribed a non-selective beta-blocker within the 3 months leading up to the audit date. | Patients aged ≥ 18 on the audit date with a Read code for asthma at least 3 months before the audit date and no Asthma resolved code. |
| P8 | Prescription of a long-acting beta-2 agonist inhaler (excluding combination products with inhaled corticosteroid) to a patient with asthma who is not also prescribed an inhaled corticosteroid. | Patients who have not been prescribed an inhaled corticosteroid within the 3 months leading up to the audit date. | Patients aged ≥ 18 on the audit date with a Read code for asthma at least 3 months before the audit date and no Asthma resolved code, who have been prescribed a long-acting beta-2 agonist inhaler (excluding combination products with inhaled corticosteroid) within the 3 months leading up to the audit date. |
| Preventing heart failure | | | |
| P9 | Prescription of an oral NSAID to a patient with heart failure. | Patients prescribed an oral NSAID within the 3 months leading up to the audit date. | Patients aged ≥ 18 on the audit date with a Read code for heart failure at least 3 months before the audit date. |
| Preventing acute kidney injury | | | |
| P10 | Prescription of an oral NSAID to a patient with chronic renal failure (eGFR <45) | Patients prescribed an oral NSAID within the 3 months leading up to the audit date. | Patients aged ≥ 18 on the audit date an eGFR <45 at least 3 months before the audit date. |
| Medication monitoring indicators | | | |
| Preventing liver damage and development of neutropaenia | | | |
| M1 | Prescription of methotrexate without both a recent full blood count and a recent liver function test | Patients receiving methotrexate for at least three months who have not had a recorded full blood count and/or liver function test within the previous three months |  |
| Preventing hypo and hyperthyroidism | | | |
| M2 | Prescription of amiodarone without a thyroid function test | Patients receiving amiodarone for at least six months who have not had a thyroid function test within the previous six months |  |

NSAID = non-steroidal anti-inflammatory drug; NOAC = novel oral anticoagulant; eGFR = estimated glomerular filtration rate; ACEI = angiotensin converting enzyme inhibitor; ARB = angiotensin receptor blocker. ^1^NOAC: apixaban or dabigatran or rivaroxaban; ^2^Antiplatelet drug: aspirin or clopidogrel or prasugrel or ticagrelor; ^3^Antiplatelet drug excluding aspirin: clopidogrel or prasugrel or ticagrelor; ^4^Peptic ulceration: this includes upper gastrointestinal bleed. It does not include peptic ulcer surgery, gastritis, duodenitis or oesophageal varices; ^5^Ulcer-healing drug: this includes the proton-pump inhibitors and H2-antagonists. It does not include Misoprostol, Sucralfate or Bismuth.
