# Supplementary material for: Evaluation of a pharmacist-led actionable audit and feedback intervention for improving medication safety in UK primary care: An interrupted time series analysis
Source: PLoS Med. 2020 Oct 13;17(10):e1003286. doi: 10.1371/journal.pmed.1003286 (PMC7553336; doi:10.1371/journal.pmed.1003286)
Supplement: Attachment — Submitted filename: 20200709 PLOS medicine response letter (2nd revision).pdf [file pmed.1003286.s008.pdf]

Response to reviews and editors' requests

PMEDICINE-D-19-04218 (Peek et al., "Impact of a pharmacist-led actionable audit and feedback intervention to improve medication safety in primary care: interrupted time series analysis")

We would like to thank the reviewers and editors for the feedback on our manuscript. Below are our responses to specific comments.

Requests from the editors:

E.R1.1. Please check the Dryad code for data access, which we were unable to access.

We have decided to switch to Mendeley Data, where the data have now been publshed at https://doi.org/10.17632/ps8jwmmnkv.1.

E.R1.2. Please adapt the title slightly to "... in UK primary care ...". As an alternative, you might wish to add "in Manchester, UK" to the segment of the title before the colon.

Thank you. We have opted for the shorter "… in UK primary care …".

E.R1.3. Please make that "43" in the abstract.

Agreed and changed.

E.R1.4. Please adapt the end of the "Methods and findings" subsection of your abstract so that the final sentence begins "Study limitations include ..." or similar. The quoted limitations could include the fact that practices were not randomized, for example, and the possibility that unmeasured confounders influence your findings.

Agreed and changed.

E.R1.5. At the end of p.4 of the PDF, please make that "Our objective was to evaluate".

Thank you. We have changed this.

E.R1.6. On p.9 of the PDF, you present various "significant reductions", but we note that not all the point estimates are significant at the 5% level. Please modify the text as appropriate.

We have revised the wording to better reflect that the difference in inadequate blood-test monitoring was longer significant after 12 months.

E.R1.7. Please signpost the discussion of limitations in the main text with "This study had some limitations ..." or similar.

Thank you – we have signposted this now.

E.R1.8. As in the abstract, you may wish to note the possible influence of unmeasured confounders on your findings.

Indeed. We have added this to the Discussion.

E.R1.9. On p.12 of the main text, please make that "... it is also possible that some".

Thank you – we have changed this.

E.R1.10. Please remove the mention of funding at the end of the main text. This information will appear in the metadata upon publication, via information in the submission form.

Thank you, we have changed this.

E.R1.11. We ask you to review the full paper to ensure that the wording does not imply that causality has been established in this research design. For example, at the top of p.11 of the PDF there is the phrase "... no previous studies have evaluated the effects ...", in which "impact" could be substituted. The author summary section also includes similar phrasing, and there is similar wording at the end of p.9 and on p.10 of the PDF ("12-month effect").

Thank you. We have made a substantial number of small changes throughout the manuscript to avoid the suggestion of causality.

E.R1.12. Please update reference 38.

Thank you. The reference has been updated.

E.R1.13. The TREND and TIDieR a_achments both contain page numbers, which we ask you to remove (as page numbers generally change upon publication). In the checklists, please refer to individual items by section (e.g., "Methods") and paragraph number.

We have replaced page numbers with relevant section references in both checklists.

E.R1.14. Please refer to the attachments in your methods section (e.g., "See S1_TREND_Checklist").

We now refer to both checklist at the start of the methods section.

***** Reviewer #1 (statistical review)**

No further comments.

***** Reviewer #3:**

R3.1. The major issue with the evaluation is stating that the intervention resulted in a change when that change is statistically insignificant. I had thought non-statistically significant changes would not be even mentioned in the abstract or described in words and well as figure. In the abstract the way the following is written it could be misread and readers might think there was a reduction at 12 months where there wasn't - "The rate of inadequate blood-test monitoring (composite of 2 indicators) reduced by 22.0% (95% CI, 0.2% to 50.7%, p=0.046) at 24 weeks and by 23.5% (95% CI, -4.5% to 61.6%, p=0.127) at 12 months." The authors are clearer in the discussion when they write "inadequate blood-test monitoring there was a decrease at 24 weeks, but not so at 12 months."

We agree that this could be misread as a significant reduction and have therefore rephrased this as follows: "the change at 12 months (23.5%) was no longer significant (95% CI, -4.5% to 61.6%, p=0.127)".

R3.8. Thank you for being consistent when describing the result times. "We agree and now use 4 weeks, 12 weeks, 24 weeks, and 12 months consistently." - it seems a little strange to choose to use a mix of weeks and months.

Thank you. We have considered the alternative (using "52 weeks" instead "12 months") but felt that it would not improve the readability of the paper and therefore decided against it. We agree that neither solution is perfect.

Additional comments related to changes made to the original paper:

R3.11. in the "What do these findings mean?" section at the start of the paper the authors state

"Electronic, actionable audit and feedback interventions can provide long-term improvements to medication safety in general pracJce - but only when they are delivered on an ongoing basis."

I am not sure the phrase "but only" can be justified from the findings of this study.

Agreed. We have removed “but only”.

R3.12. In the introduction (top of page 4) the following phrase "means that medication errors in primary care contribute to more than seven times more avoidable deaths than secondary care (627 versus 85)" has the more in it twice making it difficult to read. Perhaps "means that avoidable deaths in primary care due to medication errors are seven times more likely than in secondary care (627 versus 85)" is clearer.

Agreed and corrected, thank you.

R3.13. Page 11 in discussion "after an intensive review of patents" should be "after an intensive review of patients"?

Indeed. Corrected, thank you.

R3.14. Page 12 in discussion "The main thread to validity is bias" should be "The main threat to validity is bias"

Done, thank you.
