# Supplementary material for: Evaluation of a pharmacist-led actionable audit and feedback intervention for improving medication safety in UK primary care: An interrupted time series analysis
Source: PLoS Med. 2020 Oct 13;17(10):e1003286. doi: 10.1371/journal.pmed.1003286 (PMC7553336; doi:10.1371/journal.pmed.1003286)
Supplement: Attachment — Submitted filename: PLOS medicine Review TD.docx [file pmed.1003286.s006.docx]

**Abstract:**

- The authors report a reduced variation in the prevalence of targeted outcomes. It is not entirely clear how variation was calculated, but if it is a simple comparison of ranges, this is problematic because ranges are vulnerable to outliers. The amount of variation is also not reported in the results of the main manuscript. I suggest to drop variation in prevalence from the abstract and report variation in effect instead.

**Methods**

Description of the intervention

- In the description of the intervention, please clarify whether pharmacists were all newly employed pharmacists or had established roles
- The phrase “Dashboard content for each indicator was updated” could be misunderstood. Please, clarify that what you presumably mean is that the figures/numbers of patients triggering were updated (rather than the indicators themselves or the guidance around them)

Outcome measures

- Although it can be inferred from what is written, it would help the reader if it was explicitly stated that the patient cohort in which the outcomes were evaluated was a dynamic one, i.e. the individual patients and the numbers of patients included in the denominator could change from one audit date to the next.
- There is inconsistent language in the manuscript to describe “denominator” patients who are at risk of the outcomes. I suggest to consistently refer to those patients as having “risk factors for” or being “at risk of” potentially hazardous prescribing or inadequate monitoring.
- Suggestions for improved description of outcome measures (the denominator description varies in the manuscript between text and tables): “Our primary outcome measures were prevalence of exposure to a) any potentially hazardous prescribing (10 indicators) and b) any inadequate blood-test monitoring (2 indicators) among patients with risk factors for such prescribing and monitoring (as defined by individual indicators), respectively. These outcomes were assessed through two composite measures, defined as the sum of all patients who triggered one or more prescribing (monitoring) indicators divided by the sum of all patients with risk factors at a given audit date. Secondary outcome measures included prevalence of ongoing (existent for 30 or more days) and new (arisen within the previous 30 days) exposure to any potentially hazardous prescribing and any inadequate blood-test monitoring, and the prevalence of the twelve indicators individually. In each practice, outcomes were assessed every 4 weeks during 24 months before and 12 months after the practice started the intervention. “

Statistical methods

- It seems problematic that outcome measures are defined as prescription in previous 3 months while outcomes are assessed in monthly intervals. I would expect large amount of autocorrelation. It is essential that this is taken into account in the statistical analysis.

**Results**

- Was the start date controlled by the practice or the research team? What is the significance of the start date (e.g. is this about early versus late adopters as per diffusion of innovation ….?). Please, clarify.
- Practices started the intervention over the course of more than a year. Please, clarify what marked the start date of the intervention in each practice.
- Table 1: the percentages reported appear to have different denominators. Please, explain in column 1 what the denominator is (e.g. whether it is patients with risk factors for medication hazards or all registered patients).
- Table 2: I suggest to include a column detailing numbers of patients in numerator and denominator at baseline. This is important for the reader to be able to determine the relative contribution of individual indicators to the overall composite.
- Table 2: It seems that in the case of outcome 9, the point estimate for the change in prescribing at 52 weeks was higher than at 24 weeks but the confidence interval was wider so that the result was no longer significant. Do the authors have an explanation for this? Might there be a mistake?

**Conclusion**

- For the same reasons as stated in the abstract I recommend to drop the aspect of reduced variation if this is based on a comparison of ranges alone.
