# Supplementary material for: Evaluation of a pharmacist-led actionable audit and feedback intervention for improving medication safety in UK primary care: An interrupted time series analysis
Source: PLoS Med. 2020 Oct 13;17(10):e1003286. doi: 10.1371/journal.pmed.1003286 (PMC7553336; doi:10.1371/journal.pmed.1003286)
Supplement: Attachment — Submitted filename: PLOS medicine response letter 20200414 (final).docx [file pmed.1003286.s007.docx]

**Response to reviews and editors’ requests**

PMEDICINE-D-19-04218 (Peek et al., “Impact of a pharmacist-led actionable audit and feedback intervention to improve medication safety in primary care: interrupted time series analysis”)

We would like to thank the reviewers and editors for the valuable feedback on our manuscript. Below are our responses to specific comments.

Requests from the editors:

E.1. We note that you are unable share data publicly (even when de-identified, we assume). Are you able to include aggregate data in supplementary files for the benefit of other researchers, or via a publicly-accessible repository?

We have made the aggregated data available through DRYAD, <https://doi.org/10.5061/dryad.2280gb5p4>. The dataset is kept private while the manuscript is still in review, and will be made public upon publications. During this time it can be accessed at <https://datadryad.org/stash/share/hfch0VwdfBNJggOWZs4359Qyn1ztTqz9lJR7zxLIrvM>.

E.2. Noting the research design, we ask you to adopt softer wording in places where you currently assert that "the SMASH intervention reduced rates ..." and similar. For example, "Implementation of the SMASH intervention was associated with reduced rates of ..." would seem more apt.

We have adopted the softer wording as suggested, in the Abstract, Results, and Discussion.

E.3. Please substitute "this reduction" or "this apparent impact" for "this effect".

Agreed and substituted.

E.4. We suggest an amended title: "Evaluation of a pharmacist-led audit and feedback intervention for improving medication safety in primary care: an interrupted time-series analysis".

We have changed title as suggested.

E.5. Please reformat your abstract to comprise three subsections: "background"; "methods and findings"; and "conclusions". The final sentence of the "methods and findings" subsection should quote 2-3 of the study's main limitations.

We have reformatted the abstract as suggested.

E.6. To your abstract, please add aggregate demographic details for study participants; and study dates.

We have added demographic details and study dates to the abstract.

E.7. In your abstract and throughout the paper, please quote p values alongside 95% CI, where available.

We have added p-values to all 95% confidence intervals that relate to a comparison.

E.8. After the abstract, we will need to ask you to add a new and accessible "author summary" section in non-identical prose. You may find it helpful to take a look at one or two recent research papers published in PLOS Medicine to get a sense of the preferred style.

We have modified the box on p. 3 to create an author summary in the preferred style.

E.9. Early in the methods section, please state whether the study had a protocol or prespecified analysis plan, and if so add the relevant document(s) as a supplementary file, referred to in the text. Please highlight analyses that were not prespecified.

A research protocol was submitted to the UK Health Research Authority and approved in September 2015. We have added the protocol as supplementary file to the revised manuscript. The protocol described the intervention, the design of the study, statistical analysis methods, duration of follow-up (12 months), and sample size calculation but did not specify the post-intervention periods shorter than 12 months (4 weeks, 12 weeks, and 24 weeks) at which we assessed the effect of the intervention. This has now been explained in the **Statistical Analysis** section in the *Methods*.

E.10. We note that you mention the TREND statement in the methods section. Please add a completed checklist for the most appropriate reporting guideline, which may well be TREND, as a supplementary document, referred to in your methods section. In the checklist, please refer to individual items by section (e.g., "Methods") and paragraph number rather than by page or line numbers, as the la]er generally change in the event of publication.

A completed TREND statement has been added as supplementary file.

E.11. Please avoid claims of "the first" and the like, noting one instance in your discussion section, and where necessary please add "to our knowledge" or similar.

We have made the suggested changes.

E.12. We ask you to revisit the "strengths and limitations" section, aiming to correct an apparent imbalance in favour of the former component. For example, where you note the absence of randomization, the possible influence of unmeasured confounders could be quoted.

We have revised the “strengths and limitations” section to provide a more balanced account.

E.13. Where you mention the word "trend", please state the statistical basis for this; or substitute a different word or phrase, e.g. "apparent reduction".

This might be a misunderstanding. We only use the term “trend” to refer to the development of a given quantity over time, and not to refer to a difference between quantities. In particular, we compared the observed post-intervention rate of each indicator to the extrapolated pre-intervention trend of that same indicator – regardless of whether there was decrease, increase, or no change over time before the intervention. For this reason, we have kept the term “trend”.

E.14. Please revisit your reference list to ensure that all citations meet journal style. All italics and boldface text should be converted to plain text; and where appropriate, 6 authors names should be listed, followed by "et al.".

We have revisited the reference list to ensure that it adheres to the given specifications. We have also remove to duplicate references.

E.15. Please ensure that all references have full access details, noting reference 20, for example.

We completed details for references 19 (was 20), 38 (was 40), and 46 (was 37).

***** Reviewer #1 (statistical review)**

R1.1. Abstract - Results: I suggest that you start by providing the rates at baseline ("At baseline, 95% of practices had rates…"), before presenting your results. This will allow the reader to understand the starting point, and realise what the percentage reductions that are mentioned are relative to in a more "chronological" way.

We have changed the abstract as suggested by the reviewer.

R1.2. Statistical analysis: Could you please provide more information on your sample size calculation? Please summarise in a few words the reference levels for which you use reference 6.

Based on the study by Stocks et al. (reference 6) we assumed a baseline prescribing risk of 6% with the intervention reducing it to 4.5%. This has now been clarified in the manuscript, on p. 7.

R1.3. Also please clarify what you mean by a before-after study? Is it effectively a change in percentage? What is your estimated population size of a GP?

Yes indeed, for the sample size calculation we assumed a change in percentage from 6% to 4.5%, with a random effect for the observed reduction. In terms of population size, we assumed to have 2000 patients across 30 practices (the average list size per practice is much higher but most patients would not be relevant for our analysis). We have explained this now on p. 7.

R1.4. Finally, 88.3% power is slightly unusual. Could you please clarify?

This number stems from the fact that we calculated the sample size using assumptions on numbers (patients and practices) and not the other way around. At the time we assumed that we would be able to recruit at least 30 practices.

R1.5. Statistical analysis: the sample size calculation is 30 GPs. But you include 43 GPs. Please could you clarify why?

This was a real-world evaluation that was undertaken on the back of implementing the intervention in Salford, UK. That implementation was always meant to be permanent. At the time of conducting the sample size calculation, we assumed that it would be possible to implement the intervention in at least 30 practices. However, eventually all 43 eligible practices wanted to participate, and hence we decided to implement the intervention in all those 43 practices. They still use it.

R1.6. Results: you mention that "the rate of [] prescribing reduced significantly more after introduction of the SMASH intervention compared to extrapolations". Does this mean that you compare the projected rate of 12 months, using data from the pre-intervention period, to the observed rate at 12 months? Or effectively is it the case that you are comparing the observed rates to the rates at baseline?

We compared the observed post-intervention rates to the extrapolated pre-intervention trends over time, which were assumed to be the expected post-intervention rates had the intervention not taken places. We have changed the wording of the **Statistical Analysis** section in the *Methods* and the first part of the *Results* to better reflect this.

***** Reviewer #2:**

*Abstract:*

R2.1. The authors report a reduced variation in the prevalence of targeted outcomes. It is not entirely clear how variation was calculated, but if it is a simple comparison of ranges, this is problematic because ranges are vulnerable to outliers. The amount of variation is also not reported in the results of the main manuscript. I suggest to drop variation in prevalence from the abstract and report variation in effect instead.

Thank you for raising the point. We acknowledge the methods are complicated, and they needed to be, because we need to account for practice variation in an interrupted time series design. Using meta-analysis random-effects methods we quantify the heterogeneity in the effect, across practices, using an advanced random-effects model (Bootstrapped DerSimonian-Laird). So the reported variation is a simple range and should be robust against outliers. The variation between practices and its reduction over time is discussed both in the abstract, and in the main text on p. 9 (this might have been overlooked by the reviewer).

*Methods*

*Description of the intervention*

R2.2. In the description of the intervention, please clarify whether pharmacists were all newly employed pharmacists or had established roles

Some pharmacists had existing roles in general practice and others were recruited into similar roles for practices where no pharmacist was employed previously. This has now been clarified in the **Intervention** section of the *Methods* (p. 6).

R2.3. The phrase “Dashboard content for each indicator was updated” could be misunderstood. Please, clarify that what you presumably mean is that the figures/numbers of patients triggering were updated (rather than the indicators themselves or the guidance around them)

Indeed only the numbers and graphs were updated. Thank you for picking this up, it was indeed ambiguous. We have rephrased the relevant sentence in the *Methods*.

*Outcome measures*

R2.4. Although it can be inferred from what is written, it would help the reader if it was explicitly stated that the patient cohort in which the outcomes were evaluated was a dynamic one, i.e. the individual patients and the numbers of patients included in the denominator could change from one audit date to the next.

We agree that this is an important point and have now made this explicit.

R2.5. There is inconsistent language in the manuscript to describe “denominator” patients who are at risk of the outcomes. I suggest to consistently refer to those patients as having “risk factors for” or being “at risk of” potentially hazardous prescribing or inadequate monitoring.

We have eliminated this inconsistency by using the phrases “having one or more risk factors for being exposed” and the shorter “having risk factors for” throughout the manuscript.

R2.6. Suggestions for improved description of outcome measures (the denominator description varies in the manuscript between text and tables): “Our primary outcome measures were prevalence of exposure to a) any potentially hazardous prescribing (10 indicators) and b) any inadequate blood-test monitoring (2 indicators) among patients with risk factors for such prescribing and monitoring (as defined by individual indicators), respectively. These outcomes were assessed through two composite measures, defined as the sum of all patients who triggered one or more prescribing (monitoring) indicators divided by the sum of all patients with risk factors at a given audit date. Secondary outcome measures included prevalence of ongoing (existent for 30 or more days) and new (arisen within the previous 30 days) exposure to any potentially hazardous prescribing and any inadequate blood-test monitoring, and the prevalence of the twelve indicators individually. In each practice, outcomes were assessed every 4 weeks during 24 months before and 12 months after the practice started the intervention. “

Thank you very much. We have made this change.

*Statistical methods*

R2.7. It seems problematic that outcome measures are defined as prescription in previous 3 months while outcomes are assessed in monthly intervals. I would expect large amount of autocorrelation. It is essential that this is taken into account in the statistical analysis.

Thank you for this point. We need to highlight that we are using established clinically meaningful indicators, in which 3-month windows are generally used. This can be problematic for certain analyses, for example if we wished to identify “spikes” for service improvement and review purposes, but we do not think it poses a limitation in this context. Effectively, the signal is somewhat smoothed for these indicators, which can be seen as an advantage in this context. We think the reviewer means autoregressive errors (correlated errors over time), rather than autocorrelation (correlation of the signal with a delayed version of itself), but we have used methods that account for both of these issues (Newey–West estimator) and we found no differences when we incorporated autocorrelation assumptions.

*Results*

R2.8. Was the start date controlled by the practice or the research team? What is the significance of the start date (e.g. is this about early versus late adopters as per diffusion of innovation ….?). Please, clarify.

General practices received the intervention at different points in time, depending on the availability of trained pharmacists to help deliver this within the practices. Once a pharmacist was available, the practice controlled the precise date at which they would start the intervention. This has now been clarified under **Study design** in the *Methods* (p. 5). We reported the spread of start dates in the *Results* to give readers insight into the risk of confounding by secular trends. There was no intention to speculate on differences between early, middle, and late adopters, and for this reason we have removed the statistical comparison between groups from Table 1.

R2.9. Practices started the intervention over the course of more than a year. Please, clarify what marked the start date of the intervention in each practice.

See above at R2.8.

R2.10. Table 1: the percentages reported appear to have different denominators. Please, explain in column 1 what the denominator is (e.g. whether it is patients with risk factors for medication hazards or all registered patients).

It is the number of patients with risk factors. This has now been clarified directly beneath the table.

R2.11. Table 2: I suggest to include a column detailing numbers of patients in numerator and denominator at baseline. This is important for the reader to be able to determine the relative contribution of individual indicators to the overall composite.

We agree and have added this column.

R2.12. Table 2: It seems that in the case of outcome 9, the point estimate for the change in prescribing at 52 weeks was higher than at 24 weeks but the confidence interval was wider so that the result was no longer significant. Do the authors have an explanation for this? Might there be a mistake?

No, we checked this result and it is correct. For this indicator the standard error (i.e. heterogeneity between practices) was higher at 12 months, and therefore the decrease in point estimate was no longer significant. The explanation is that the majority of practices continued to reduce prescription of NSAIDs to patients with heart failure but there were also practices where it crept up after 6 months. This led to high heterogeneity in the meta-analysis at 12 months.

We have added to the Results on p. 10: “For the latter type of hazardous prescribing, the average across all practices still decreased at 12 months but this decrease was no longer significant due to increasing variation between practices.”

*Conclusion*

R2.13. For the same reasons as stated in the abstract I recommend to drop the aspect of reduced variation if this is based on a comparison of ranges alone.

As explained in our response to R2.1, we have re-introduced the reporting of variation in prevalence that had inadvertently dropped out of the original manuscript. Therefore we have kept the statement about reduced variation.

***** Reviewer #3:**

*Major issues*

R3.1. The major issue with the evaluation is stating that the intervention resulted in a change when the confidence intervals of the estimated effect size cross zero. For example in the abstract (page 8); results section 2^nd^ sentence is the following statement: “The rate of inadequate blood-test monitoring reduced by 22.0% (95% CI, 0.2% to 50.7%) at six months and by 23.5% (95% CI, -4.5% to 61.6%) at 12 months.”

Given the confidence interval of the 12 months figure crosses zero the reduction is not statistically significant (at a 0.05 significance level).

This is repeated in the body of the paper in the Rates of potentially hazardous prescribing and inadequate bloodtest monitoring section, on page 15: “The rate of inadequate blood-test monitoring reduced significantly more compared to the extrapolated pre-intervention trend with an absolute difference at four weeks of -2.40 (95% CI, -4.53 to 0.08), twelve weeks -2.594 (95% CI, -4.70 to -0.13), 24 weeks -2.824 (-5.15 to -0.18), and 52 weeks -2.85 (95% CI, -5.68 to 0.71), corresponding to relative reductions of 17.8% (95% CI, 0.5 to 41.4), 19.6% (95% CI, 0.8 to 44.3), 22.0% (95% CI, 0.2 to 50.7%), and 23.5% (95% CI, -4.5 to 61.6) respectively.”

Again the confidence intervals for the absolute rates cross zero in the 4 week and 52 week figures so there is no statistically significant reduction (at a 0.05 significance level) for these two times. It would be helpful if the authors could explain how the relative reduction at 4 weeks is statistically significant when the absolute at 4 weeks is not.

In light of these comments the statements in the conclusion would need to be reviewed as there is no statistically significant reduction in the blood monitoring (95% confidence) at 12 months following the implementation of the intervention.

We agree with the reviewer and have revised the conclusions regarding the reduction at 12 months accordingly (p. 2 [Abstract]; p. 14 [Conclusion]).

The reported lower bound (0.5) of the relative reduction in inadequate blood-test monitoring at four weeks was incorrect and should have been -0.5. At this point in time, the reduction was not statistically significant. We are grateful to the reviewer for spotting this and have corrected it in the manuscript (p. 9).

R3.2. The methods describe describes the practice-level analysis being done at 4, 12, 24 and 52 weeks but only the 52 week results are presented (in the last paragraph of the results section and as the supplementary figures 2 and 3). Related to this, an interpretation of the weaker correlation between pre and post intervention trends in the composite measure of potentially hazardous prescribing compared to the composite measure of blood monitoring would be useful to make it clearer why this statistic is being reported.

We have now provided all the forest plots in the appendix.

R3.3. In the discussion, there is a statement at the top of page 11 "In other words, our intervention appeared to not only accelerate the pre-intervention trend in practices where it existed but also initiated it where it was absent." No evidence or analysis that showed those practices with higher levels of potentially hazardous prescribing prior to the intervention did not have declining trends leading up to the intervention was provided. Was that analysis done but not provided/reported?

Agreed that this statement was not supported by analyses presented in the manuscript, and we have therefore decided to remove it.

R3.4. It might be useful to the reader if the authors were able to suggest reasons for the difference in outcome for the difference measures using the results from the qualitative analysis. Understanding why some measures are unchanged by this intervention (e.g. prescribing beta-blockers to patients with a diagnosis of asthma) would help others when they replicate this initiative as it might mean some types of potentially hazardous prescribing require a totally different type of intervention in order to have an effect or might be that an adjustment of this intervention is what is required.

The qualitative study was conducted during the roll-out of the intervention, before the results from the quantitative evaluation were known. Therefore we feel it would be speculative to try to find explanations for these findings in the results from the qualitative study.

Through later discussions with some of the participating pharmacists a few things were noted. First, in some instances, GPs are hesitant to halt prescribing started by other specialist practitioners (e.g. psychiatrists or optomotrists) which might explain the lack of change in prescriptions of beta-blockers. Second, there exist instances where no suitable alternative treatment is available for the patient in question, and therefore no changes are made to a potentially hazardous prescription.

We have added these two potential explanations to the *Discussion*.

R3.5. The 12 PINCER indicators were selected based on their prevalence nationally and in Salford; was it low prevalence or high prevalence (or some other criteria) used to select the 12. Readers might benefit from seeing the PINCER measures not selected and the prevalence of all of them.

We left out indicators with a very low prevalence because it was felt that for these indicators, the benefits of implementating the intervention would not outweigh the effort. This has now been clarified on p. 5 in the section **Indicators** in the *Methods*. We refer to the papers by Stocks et al. (reference 6) and Akbarov et al. (reference 21) for an overview of all indicators and their prevalence in Salford and nationally.

R3.6. The paper describing the evaluation of the intervention follows the TREND style. A more structured description of the intervention might help the reader assess whether the intervention could be replicated in their own setting e.g. the template for intervention description and replication (TIDieR). For example, replicating this in other areas it would be useful to know whether the medication reviews were done with or without the patient present.

Thank you for this suggestion. We have completed the template for intervention description and replication (TIDieR) and added this as a supplementary file.

*Minor issues*

R3.7. In Table 1 please provide an explanation for the p-value in the final column - what measures do the p-values relate to?

These p-values related to differences between the columns, assessed through Chi-squared / ANOVA testing. On reflection we felt that these p-values were not meaningful and have decided to remove them. We have also shorted the corresponding text in the *Results* on p. 8.

R3.8. The results were measured at 4, 12, 24 and 52 weeks but sometimes the 24 week and 52 week results are described as six month and 12 month (and once the 52 weeks is described as one year). It would help if the description of the timeframe of the post-intervention analysis was consistent and accurate.

We agree and now use 4 weeks, 12 weeks, 24 weeks, and 12 months consistently.

R3.9. Results in the text sometimes have 3 significant figures, 2 are used in table 2 and 1 in supplementary table 2. It would help if the used of significant figures was consistent.

Thank you. We now use 2 significant figures throughout.

R3.10. In the methods section, bottom of page 6, the statement "All outcomes data were collected by interrogating primary care electronic health records" is this a separate extract/database to the SIR database? If it is separate would it be possible to provide additional details sufficient to allow replication by other researchers?

This relates to the SIR database, not a separate database. We have amended the wording to better reflect this: “All outcomes data were collected by interrogating primary care electronic health records from the SIR data warehouse for relevant diagnoses, prescriptions, and laboratory test results.”
